# Implication of the Pro12Ala polymorphism of the *PPAR-gamma 2 *gene in type 2 diabetes and obesity in the French population

**DOI:** 10.1186/1471-2350-6-11

**Published:** 2005-03-22

**Authors:** Maya Ghoussaini, David Meyre, Stéphane Lobbens, Guillaume Charpentier, Karine Clément, Marie-Aline Charles, Maïté Tauber, Jacques Weill, Philippe Froguel

**Affiliations:** 1 CNRS UMR 8090, Institute of Biology of Lille, Pasteur Institute, Lille, France; 2 Endocrinology-Diabetology Unit, Corbeil-Essonnes Hospital, France; 3 EA3502, Hôtel-Dieu Hospital, Paris, France; 4 INSERM U258, Paul Brousse Hospital, Villejuif, France; 5 INSERM U563, Children's Hospital, Toulouse, France; 6 Pediatric Endocrine Unit, Jeanne de Flandre Hospital, Lille, France; 7 Genomic Medicine and Genome Centre, Hammersmith Campus, Imperial College London, UK

## Abstract

**Background:**

The Pro12Ala Single Nucleotide Polymorphism (SNP) of the Peroxisome Proliferator-Activated Receptor gamma 2 (*PPAR-gamma 2*) has been associated with insulin resistance and type 2 diabetes (T2D) and also inconsistently with obesity. The aim of this study was to evaluate the impact of this SNP with regards to T2D and childhood and adult obesity in the French Caucasian population.

**Methods:**

We conducted three independent case/control studies encompassing 2126 cases and 1124 controls.

**Results:**

We found a significant association between *PPAR-gamma 2 *Pro12Ala SNP and T2D (p = 0.04, OR = 1.37), which was stronger when the T2D cohort was stratified according to the obesity status (p = 0.03, OR = 1.81 in obese T2D subjects). In contrast, there was no association between the Pro12Ala SNP and childhood and adulthood obesity. In normal glucose tolerant obese adults (but not in lean subjects), the Pro12 allele was associated with a significant increase in fasting insulin levels (p = 0.01), and in insulin resistance estimated by the Homeostasis Model Assessment of Insulin Resistance (HOMA-IR) (p = 0.003), after adjustment for age, gender and BMI. We didn't detect evidence for an interaction effect between the Pro12Ala SNP and the obesity status with respect to the HOMA-IR index in normal glucose tolerant children, but we found a borderline interaction (p = 0.06) in normal glucose tolerant adults.

**Conclusion:**

Our results showed that the Pro12Ala polymorphism is not associated with childhood or adult obesity in the French Caucasian population. In contrast, we confirm a contribution of the *PPAR-gamma 2 *Pro12 allele in the genetic risk forT2D, especially in obese subjects, where this allele worsens insulin resistanceand increases fasting insulin levels.

## Background

Insulin resistance is associated with obesity especially when centrally distributed [1,2]. The connection between increased adiposity and insulin resistance is still poorly understood, although recent evidence has suggested that adipose tissue-released cytokines like adiponectin, resistin, leptin and Tumour Necrosis Factor-Alpha (TNF alpha) may be contributory factors [3-6]. In this context, the activation of the adipocyte expressed nuclear receptor PPAR-γ2, by the new insulin-sensitising drugs thiazolidinediones [7,8], was suggested to increase plasma adiponectin levels, which contributed to the promotion of fatty acid oxidation and insulin sensitivity in muscle and liver [9,10] and also to the inhibition of the expression of TNF-α and resistin [4,11-13].

There is a frequent non synonymous (cytosine to guanine) single nucleotide polymorphism (SNP) in *PPAR-γ2 *exon 2 [14]. This variation results in a Proline to Alanine substitution at the codon 12, which has been found to modulate the transcriptional activity of the gene [15,16]. Several studies have reported an association between Ala12, increased insulin sensitivity and a reduced risk of type 2 diabetes [17-20] which has been supported by meta-analysis [17]. However, results assessing the risk of the Pro12Ala in obesity were controversial [21,22].

We previously studied the effect of this variant in a rather limited sample of French Caucasians [23] and found no association between the Pro12Ala and T2D or obesity in this population. Given the modest impact of the Pro12Ala in the T2D risk [17], the previous study may have probably lacked the statistical power for replication. In order to achieve a definite conclusion about this gene variant and "diabesity" in French Caucasians, this present study has analysed large sample sets representative of the condition and as well as controls (altogether 2126 cases and 1124 controls). We designed three independent case/control studies to assess the presence of any association between the Pro12Ala SNP and T2D, childhood obesity or adulthood obesity. In addition the putative effect of this SNP on insulin sensitivity and insulin secretion indexes in normal glucose tolerant (NGT) lean subjects and in NGT obese subjects was evaluated, and as well any possible interaction between the SNP and adiposity on insulin sensitivity, as recently reported in the US population, was also assessed [24].

## Methods

### Subjects

The DNA samples were extracted from EDTA whole-blood samples using Puregene Kit (Gentra, Minneapolis, M N). Six samples sets were used for association studies and for analysis of variance of phenotypic traits: Association studies with childhood obesity were performed using a set of 195 unrelated lean children from the "Fleurbaix-Laventie Ville Santé" and a set of 396 unrelated obese children chosen from the cohort of 554 obese children available. The pool of obese children used in association studies was constituted by a first set of 278 unrelated obese children collected from 278 pedigrees with at least one obese child at the CNRS-Institut Pasteur Unit and at the Jeanne de Flandres Hospital in Lille, a second set of 90 unrelated obese children recruited at the Children's Hospital, Toulouse [25], and a third set of 28 unrelated obese children recruited through the "Fleurbaix-Laventie Ville Santé" study. Children with a BMI greater than the 97^th ^percentile of BMI for age and sex reported on the tables of Rolland-Cachera *et al*. [26] (French general population) were defined as obese as recommended by the European Childhood Obesity Group (ECOG) [27]. From the 376 lean children available in the Fleurbaix-Laventie study, only 195 unrelated children were included as control, since all children with at least one obese sibling were excluded.

Adult obesity association studies were performed using two sets of adult obese patients: the first constituted by 450 moderately obese adults and the second constituted by 652 morbidly adult obese patients. These individuals were collected at the Department of Nutrition of the Hôtel Dieu Hospital in Paris or at the CNRS-Institut Pasteur Unit in Lille. Obesity status was defined as BMI ≥ 30 in adults. We used as control group a set of 611 unrelated non obese and non diabetic subjects recruited at the CNRS-Institut Pasteur Unit in Lille (N = 345) and through the "Fleurbaix-Laventie Ville Santé" study (N = 266) [28].

Association studies with T2D were performed using a set of 628 type 2 diabetic subjects. Diabetic state was informed by a fasting and/or glucose-tolerance test according to the WHO 1999 criteria. Patients are part of a publicity advertised campaign for "200 families to overcome diabetes", of CNRS-Institut Pasteur de Lille recruitment (N = 365) or recruited at the Sud Francilien Hospital in Corbeil-Essonnes (N = 263). We used a group of 318 non diabetic unrelated spouses from T2D and obesity families, aged more than 50 years old with a fasting glycaemia less than 5.6 mmol/l and recruited by a multimedia campaign at the Institut Pasteur of Lille.

Quantitative traits were calculated using normal glucose tolerant subjects selected from each of the initial sample groups previously described (Table 1). Normal Glucose Tolerance was defined by fasting glycaemia lower than 6.1 mmol/l and by a 2-hour post OGTT glycaemia lower than 7.8 mmol/l, according to the WHO 1999 criteria when available. For quantitative trait analysis, a set of 362 NGT lean children, 507 NGT obese adults and 525 NGT obese children were analysed. The 865 non obese NGT adults cohort was constituted by pooling 547 non obese NGT subjects and 318 non diabetic subjects.

Genotype-BMI interaction effect on insulin resistance was calculated separately in NGT children and adults. In each interaction test, obese and lean subjects were pooled together. The phenotypic characteristics of all the studied populations are displayed in Table 1.

### Clinical parameters

The Body Mass Index (BMI) was calculated as weight (Kg) divided by height (m) squared. The Z score of BMI was calculated using Cole's last mean square method [29]. Quantitative measurements of plasma insulin were carried out using double-antibody radio immunoassays. Serum glucose concentrations were measured using a glucose oxidase procedure. HOMA-IR and HOMA-B were calculated according to Matthews et al [30].

### Genotyping

Genotyping of the Pro12Ala of the PPAR gamma 2 gene was performed using the Taqman Allelic discrimination (AD) Assay (Applied Biosystem). The Taqman genotyping reaction was amplified on a GeneAmp PCR system 9600 (95°C for 10 minutes, followed by 40 cycles of 92°C for 15 seconds, and 60°C for 1 minute), and fluorescence was detected on an ABI Prism 7900 sequence detector (Applied Biosystem). The mix used in the Taqman experiment contained 2.5 μl of the master mix, 0.25 μl of Primers, 0.25 μl of water and 2 μl of DNA with a concentration of 10 ng/μl.

### Statistical analysis

Statistical analysis were carried out using SPSS 10.0 program (SPSS, Chicago, IU, USA). The Pro12Ala variant was complied with Hardy-Weinberg proportions. Fisher's exact test was applied to compare allelic frequencies between case and controls [31]. Quantitative traits were studied using the general linear model (GLM), taking into account gender, age, BMI and genotype effect (Pro12Pro *versus *Pro12Ala/Ala12Ala-dominant model). The BMI quantitative trait was adjusted only by gender and age. The genotype-BMI and genotype-obesity status interaction effects on insulin resistance were also tested using a GLM procedure. HOMA-IR was the dependant variable. Age, gender, BMI (or obesity status), and Pro12Ala polymorphism were the explicative factors. A term of interaction BMI*Pro12Ala, or obesity status*Pro12Ala was introduced. The Z score of BMI was used in analyses including obese children.

## Results

### Phenotypic characteristics of the studied populations

Phenotypic characteristics of the six populations are shown in Table 1. Compared to lean children, obese children have higher fasting glycaemia and fasting insulinemia (11.88 UI/l *versus *5.77 UI/l, p < 0.001). Obese adult subjects have higher glycaemia and fasting insulinemia (16.11 UI/l *versus *5.77 UI/l, p < 0.001) than non obese subjects. T2D subjects have higher BMI and fasting insulinemia than non diabetic subjects (11.18 UI/l *versus *7.40 UI/l, p < 0.001).

### Effect of the Pro12Ala SNP on obesity

The genotypic distributions of the Pro12Ala did not significantly deviate from the Hardy Weinberg equilibrium in any of the six groups of subjects.

There was no significant difference in the allelic frequencies between the four analysed sample sets as shown in Table 2, ruling out any association between this SNP and severe childhood or adulthood obesity in the French Caucasian population.

### Effect of the Pro12Ala SNP on T2D

**Figure 1:**
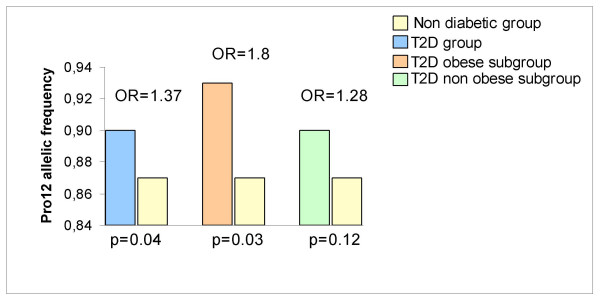
Allelic frequency of the Pro12Ala polymorphism in non diabetic and type 2 diabetic subjects (T2D) stratified according to obesity status.

As previously shown in other ethnic groups, the "at risk" Pro allele was modestly but significantly more frequent in T2D subjects than in non diabetic controls (0.87 in control *versus *0.90 in T2D subjects, respectively, p = 0.039, OR = 1.37, see Table 3 and Figure 1). When T2D subjects were stratified according to their obesity status (defined by a BMI = 30), the association with T2D was only significant in the obese diabetic subgroup (p = 0.030) where it was found to display a rather strong genetic risk (OR = 1.81) than the non obese diabetic subgroup (OR = 1.28, p = 0.12, Table 3, Figure 1).

### Effect of the Pro12Ala SNP on insulin sensitivity and secretion indexes in lean normal glucose tolerant (NGT) French Caucasians

The effect of the Pro12Ala variant on diabesity associated quantitative phenotypes was then assessed under a dominant model in the lean NGT controls (865 adults, and 362 children). The Ala allele was not significantly associated with fasting insulinemia, glycaemia, BMI or Z score of BMI, HOMA-IR (insulin resistance index) or with HOMA-B (insulin secretion index) in any of these two sample sets (Table 4).

### Effect of the Pro12Ala SNP on insulin sensitivity and secretion in obese NGT French Caucasians

The impact of the Pro12Ala SNP in the cohort of 1032 normal glucose tolerant obese subjects was then evaluated. In the adult subgroup (n = 507), subjects carrying the "protective" 12Ala allele showed a significant increase in their insulin sensitivity (p = 0.003), and consistently, a decrease in fasting insulinemia (p = 0.01) and of glycaemia (p = 0.04, see Table 4). In obese children (n = 525), the same trend was observed although it did not reach statistical significance. No effect on insulin secretion index (HOMA-B), Z score of BMI or BMI was found (Table 4).

### Interaction between the Pro12Ala SNP and adiposity on insulin resistance

**Figure 2:**
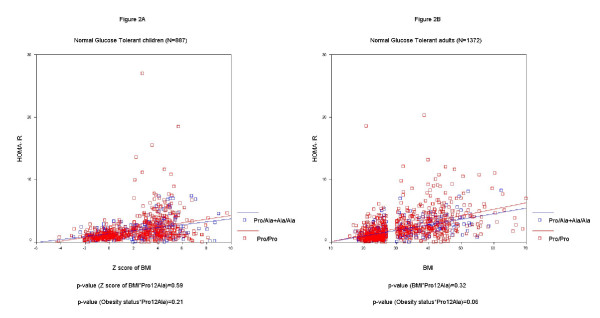
**Interaction between the Pro12Ala SNP and adiposity on HOMA-IR**. The interaction was tested using a GLM procedure. HOMA-IR was the dependant variable. Age, gender, BMI or obesity status, and Pro12Ala polymorphism were the explicative factors. A term of interaction BMI*Pro12Ala or obesity status*Pro12Ala was introduced. The Z score of BMI was used rather than BMI in analyses including obese children. (A) No evidence for interaction was found neither for Z score of BMI*Pro12Ala (p = 0.59) nor obesity status*Pro12Ala (p = 0.21) in 887 normal glucose tolerant lean and obese children (p = 0.58). (B) No evidence for interaction was found for BMI*Pro12Ala (p = 0.32) in 1372 normal glucose tolerant obese and lean adults subjects, but a borderline significant interaction was found for obesity status*Pro12Ala (p = 0.06).

The relationship between the Pro12Ala SNP, HOMA-IR and the Z score of BMI or BMI in NGT children and adults respectively was evaluated. Thus, we analysed separately 887 lean or obese NGT children and 1372 NGT adults (obese and lean together). The general linear model (GLM) analysis taking into account the continuous trait Z score of BMI or BMI supported the null hypothesis of no interaction between the Pro12Ala and the corpulence on HOMA-IR values in both ages (p = 0.59 and p = 0.32, see figures 2A and 2B). A similar GLM analysis using the obesity status binary trait (BMI ≥ 97^th ^percentile in children and BMI ≥ 30 kg/m^2 ^in adults) revealed no evidence for interaction (p = 0.21) in NGT children, but a borderline interaction effect (p = 0.06) between the Pro12Ala SNP and the obesity status with respect to the HOMA-IR index in normal glucose tolerant adults.

## Discussion

In the present study, and in contrast to what we have previously reported [23], T2D subjects were found to possess a significantly higher frequency of the Pro12 allele risk than non diabetic controls, thus supporting a role for *PPAR-γ2 *in the genetic risk for type 2 diabetes in French Caucasians. In contrast, no association with severe forms of obesity was reported in both adults and children confirming our previous finding. Interestingly, in the obese subgroup of diabetics, the Pro12 allele was found to nearly double the risk for T2D, although it has a weaker effect in lean individuals. This discrepancy might be due to an obesity-dependent effect of the "at risk" allele on insulin sensitivity. Indeed, obese adults carrying the Pro12Pro genotype were more insulin resistant than subjects carrying at least one Ala12 allele, with a similar trend seen in obese children, although no such effect was found in lean controls.

Li et al [24] reported that the detrimental effect of the Pro12 allele on insulin sensitivity was stronger in adult subjects with the highest BMI in the white Caucasian general population, with similar trends observed in children. However, in contrast with these data, we did not find in the entire cohorts of French adults or children formal evidence for an interaction between adiposity (evaluated by the BMI or the Z score of BMI) and the *PPAR-γ2 *Pro12Ala SNP on the variation of the insulin sensitivity index. Consistent with our results, Buzzetti et al. also failed to confirm this interaction in an Italian general population [32]. We then used the obesity status rather than the continuous trait BMI or the Z score of BMI in the analysis of interaction effect. Indeed, the BMI range observed in our samples was bimodal (due to the pooling effect of non obese and obese individuals) and highly skewed regarding a population-based cohort, suggesting that the study of a binary trait could be more relevant in our experimental design. We didn't detect any interaction in NGT children group (p = 0.21), but a borderline interaction effect (p = 0.06) between the Pro12Ala SNP and the obese status with respect to the HOMA-IR index in normal glucose tolerant adults. Our results, according to the hypothesis advanced by Li et al [24], confirmed that an obesity background could worsen the detrimental effect of the *PPAR-γ2 *Pro12 allele on insulin sensitivity in adult subjects, but not in children.

Clément's negative data with T2D in the same ethnic group [23] may highlight the fact that studies with modest sample size can fail to detect true associations. Risch et al. [33] and Lohmueller et al[34] both state that inadequate power in replication studies may contribute to the large number of non replications, hence underlining the importance of working with large enough sized populations in order to show a modest variant's effect in a multifactorial disease context. Indeed, meta-analysis are in line with our present data [17].

In our T2D population, the frequency of the protective Ala12 variant was under-represented compared with adult population controls (0.10 *versus *0.13, respectively) but is very similar with lean children's cohort (0.10 *versus *0.10, respectively). Thus, in French children, the prevalence of the "at risk" allele is surprisingly higher than in middle-aged non diabetic individuals. However, our results confirm those recently obtained by Doney et al in a Scottish cohort [35]. Indeed, they suggested that the protective effect of Ala12 against T2D and other metabolic diseases which increase the risk for premature coronary heart disease, could result in the selective recruitment of elderly Ala carriers in control populations.

The Ala12 allele resulted in a reduction in the transcriptional activity of PPAR-γ2 [15] and was associated with an increase of insulin sensitivity. Interestingly, data from murine models support these findings; Moderate reduction of PPAR-γ activity in heterozygous PPARγ-deficient mice prevented adipocyte hypertrophy, increased fatty acid combustion and decreased lipogenesis, which resulted in increased insulin sensitivity with regards to both glucose disposal and suppression of glucose production [36-38]. Moreover, these mice showed lower fasting plasma insulin, higher leptin and adiponectin serum levels [39]. Stumvoll et al. showed that alterations in the transcriptional activity of the Ala variant in adipocytes could primarily enhance insulin action on suppression of lipolysis, resulting in a decreased release of free fatty acids (FFAs) [40]. Boden et al. demonstrated that reduced availability of FFAs would permit muscle to utilize more glucose and the liver to suppress glucose production more efficiently upon insulin stimulation [41]. Taken together, these two results can explain the improvement of the insulin sensitivity as a consequence of the Ala allele.

## Conclusion

In summary, we confirm that, in the French Caucasian population, the *PPAR-γ2 *12Ala SNP allele confers a reduced risk for T2D, and decreased obesity-associated insulin resistance although it was not associated with childhood or adulthood obesity. In this regard *PPAR-γ2 *can be considered as a "diabesity" gene.

## Competing interests

The author(s) declare that they have no competing interests.

## Authors' contributions

Maya Ghoussaini and Stéphane Lobbens genotyped the Pro12Ala polymorphism of the PPAR-gamma2 gene in the studied populations. David Meyre and Maya Ghoussaini performed the statistical analyses to evaluate the Pro12Ala effect. Philippe Froguel and David Meyre have directed the study and the redaction of the article that was written by Maya Ghoussaini. DNA was provided by Guillaume Charpentier, Karine Clément, Marie-Aline Charles, Maïté Tauber and Jacques Weill.

## Pre-publication history

The pre-publication history for this paper can be accessed here:



## Figures and Tables

**Table 1:** Phenotypic characteristics of the studied populations.

Phenotypic traits	Obese Children	Lean Children	Obese Adults	Non Obese Adults	Type II Diabetics	Non Diabetic Adults
Sex (M/F)	264/290 N = 554	198/178 N = 376	357/745 N = 1102	234/377 N = 611	353/276 N = 628	123/195 N = 318
Age (years)	10.44 ± 0.16 N = 554	13.61 ± 0.13 N = 376	47.15 ± 0.41 N = 1102	51.04 ± 0.52 N = 611	59.35 ± 0.45 N = 625	62.03 ± 0.66 N = 318
BMI (kg/m^2^)	28.16 ± 0.27 N = 554	18.14 ± 0.13 N = 365	42.32 ± 0.265 N = 1102	22.95 ± 0.09 N = 604	26.84 ± 0.14 N = 616	25.69 ± 0.30 N = 318
Z score of BMI	4.10 ± 0.06 N = 554	- 0.24 ± 0.05 N = 365	-	-	-	-
Fasting glycaemia (mmol/l)	4.93 ± 0.02 N = 546	4.71 ± 0.02 N = 364	6.73 ± 0.08 N = 1056	5.03 ± 0.24 N = 571	9.45 ± 0.13 N = 611	4.95 ± 0.02 N = 318
Fasting insulinemia (UI/l)	11.88 ± 0.48 N = 491	5.77 ± 0.21 N = 362	16.11 ± 0.40 N = 1003	S.77±0.22 N = 570	11.18 ± 0.57 N = 304	7.40 ± 0.36 N = 318

Values are expressed as mean ± mean standard error.

**Table 2:** Comparison of Genotypic and Allelic Distribution of Pro12Ala polymorphism in obese *versus *non obese subjects. Case/Control studies show that the Pro12Ala polymorphism is not associated with childhood or adult obesity in the French Caucasian population (p = 0.19, p = 0.92 respectively).

Populations	PPAR genotypes			Allelic frequencies		P-value of allelic frequencies
	Pro/Pro	Pro/Ala	Ala/Ala	Pro	Ala	

Obese children (n = 396)	304 (76.8%)	84 (21.2%)	8 (2.0%)	0.87	0.13	P = 0.19 OR = 0.77 CI = [0.52–1.14]
Lean children (n = 195)	156 (77.9%)	39 (21.0%)	0 (1.1%)	0.90	0.10	
Obese adults (n = 1102)	857 (77.9%)	231 (21.0%)	12 (1.1%)	0.88	0.12	P = 0.92 OR = 1.01 CI = [0.81–1.26]
Non obese adults (n = 611)	478 (78.2%)	123 (20.2%)	10 (1.6%)	0.88	0.12	

OR: Odd Ratio and CI: Confidence Interval.

**Table 3:** Comparison of Genotypic and Allelic Distribution of Pro12Ala polymorphism in diabetic *versus *non diabetic subjects. Case/Control studies show the association of the Pro12Ala polymorphism with T2D in the French Caucasian population (p = 0.04). The stratification of diabetic subjects on the obesity status shows that the association previously found is rather due to obese T2D subgroup (p = 0.03) than non obese T2D subgroup (p = 0.12).

Populations	PPAR genotypes			Allelic frequencies		P-value of allelic frequencies
	Pro/Pro	Pro/Ala	Ala/Ala	Pro	Ala	

T2D patients (n = 628)	511 (81.4%)	113 (18.0%)	4 (0.6%)	0.90	0.10	P = **0.04 **OR = 1.37 C.I = [1.02–1.85]
T2D obese patients (n = 114)	98 (86.0%)	15 (13.1%)	1 (0.9%)	0.93	0.07	P = **0.03 **OR = 1.81 C.I = [1.05–3.13]
T2D non obese patients (n = 503)	403 (80.1%)	97 (19.3%)	3 (0.6%)	0.90	0.10	P = 0.12 OR = 1.28 C.I = [0.94–1.74]
Non diabetic subjects (n = 318)	246 (77.4%)	63 (19.8%)	9 (2.8%)	0.87	0.13	

OR: Odd Ratio and CI: Confidence Interval.

**Table 4:** Biochemical parameters of subjects according to Pro12Ala genotypes.

Populations	Genotypes	Fasting insulin (UI/l)	Fasting glucose (mmol/l)	Body Mass Index (Kg/m^2^)	Z score of BMI	HOMA-IR	HOMA-B
NGT non obese adults (n = 865)	P/P = 673	6.28 ± 0.24	4.92 ± 0.02	23.61 ± 0.14	-	1.39 ± 0.05	92.99 ± 3.44
	P/A+AA = 192	6.23 ± 0.31	4.94 ± 0.03	24.02 ± 0.26	-	1.38 ± 0.07	95.11 ± 7.19
	P-value	0.92	0.65	0.16	-	0.90	0.78
NGT non obese children (n = 362)	P/P = 291	5.39 ± 0.17	4.70 ± 0.21	18.12 ± 0.15	- 0.24 ± 0.06	1.13 ± 0.04	95.84 ± 3.47
	P/A+AA = 71	6.21 ± 0.42	4.71 ± 0.04	18.19 ± 0.28	- 0.27 ± 0.12	1.315 ± 0.09	111.58 ± 9.62
	P-value	0.06	0.67	0.84	0.84	0.06	0.62
NGT obese adults (n = 507)	P/P = 397	14.35 ± 0.60	5.30 ± 0.02	40.82 ± 0.42	-	3.41 ± 0.15	171.07 ± 7.60
	P/A+AA = 110	11.93 ± 0.72	5.19 ± 0.05	40.08 ± 0.70	-	2.75 ± 0.16	165.89 ± 17.31
	P-value	**0.01**	**0.04**	0.40	**-**	**0.003**	0.76
NGT obese children (n = 525)	P/P = 402	12.00 ± 0.59	4.90 ± 0.22	27.92 ± 0.30	4.06 ± 0.06	2.659 ± 0.14	178.71 ± 8.66
	P/A+AA = 123	10.77 ± 0.74	4.89 ± 0.42	28.55 ± 0.62	4.20 ± 0.14	2.368 ± 0.16	169.31 ± 16.48
	P-value	0.18	0.61	0.33	0.37	0.18	0.37

Data are given as mean ± mean standard error. Quantitative traits are shown according to the Pro12Ala genotypes in healthy and NGT obese French populations under a dominant model. Z score of BMI was used rather than BMI in children. Quantitative traits were studied using a general linear model procedure, taking into account gender, age, BMI and genotype effect. BMI was adjusted only by gender and age. Z score of BMI was compared according to the Pro12Ala genotypes using the T-test. Pro allele is indicated as P and Ala allele as A.
